# The impacts of pill contraceptive low-dose on plasma levels of nitric oxide, homocysteine, and lipid profiles in the exposed vs. non exposed women: as the risk factor for cardiovascular diseases

**DOI:** 10.1186/s40834-020-00110-z

**Published:** 2020-06-10

**Authors:** Zahra Momeni, Ali Dehghani, Hossein Fallahzadeh, Moslem Koohgardi, Maryam Dafei, Seyed Hossein Hekmatimoghaddam, Masoud Mohammadi

**Affiliations:** 1 Department of Biostatistics & Epidemiology, Health Faculity, Shahid Sadoughi University of Medical Sciences and Health Services, Yazd, Iran; 2 Department of Health Education & Health Promotion, Rafsanjan University of Medical Sciences and Health Services, Kerman, Iran; 3 Department of Midwifery, School of Nursing and Midwifery, Shahid Sadoughi University of Medical Sciences and Health Services, Yazd, Iran; 4 Department of Laboratory Medicine, School of Paramedicine, Shahid Sadoughi University of Medical Sciences and Health Services, Yazd, Iran; 5 Department of Nursing, School of Nursing and Midwifery, Kermanshah University of Medical Sciences, Kermanshah, Iran

**Keywords:** Oral contraceptive pills, Homocysteine, Nitric oxide, Lipid profile, Combined cohort study

## Abstract

**Background:**

Consuming oral contraceptive pills is one of the methods for preventing pregnancy worldwide. As using the pills has always caused the greatest concern for the likelihood of developing cardiovascular diseases and also given the limited conducted studies in this regard, this study was carried out to determine the impacts of low dose birth control pills on plasma levels of nitric oxide, homocysteine, and lipid profiles in the exposed vs. non exposed women as the risk factors for cardiovascular diseases.

**Methods:**

This was a combined cohort study conducted on 100 women, having the age range 20–35 years and normal menstrual cycles, referred to the health care centers in Yazd, Iran. The demographic data were obtained through face to face interviews performed by the researcher. Anthropometric indexes were measured and biochemical factors were determined by testing blood samples. Then, using SPSS 16 and statistical independent t-test and Chi- square, the data were analyzed.

**Results:**

The mean ± standard deviations of plasma levels of homocysteine, nitric oxide, cholesterol, triglyceride, Low Density Lipoprotein, and High Density Lipoprotein levels in the group consumed low dose contraceptive pills were 3.84 ± 2.35 μmol/l, 181.36 ± 90.44 μM, 180.7 ± 38.28 mg/dl, 129.82 ± 47.92 mg/ dl, 101.42 ± 30.66 mg/dl, and 56.46 ± 8.42 mg/dl, There were significant statistical differences between those consuming the pills and those not consuming the pills regarding cholesterol (*P* < 0.05).

**Conclusion:**

Consuming Low Dose contraceptive pills can increase the plasma levels of cholesterol, triglyceride, and Low Density Lipoprotein levels; i.e. this condition is called dyslipidemia. On the other hand, there were no changes in the levels of homocysteine and nitric oxide in the healthy women consuming the pills; therefore, the pills may not develop cardiovascular diseases in healthy women. Accordingly, it is recommended that the health care providers prescribe the pills for the women with cautions.

## Background

Oral contraceptive pills (OCP_s_) are one of the most common worldwide used methods for preventing pregnancy [1]; particularly, in Iran [2]. The previous generations of the pills had high doses of ethinyl estradiol which caused great concerns [3].

Nowadays, LD pills contain levonorgestrel (0.13 mg) and ethinyl estradiol (0.03 mg) [4, 5]. Since introducing OCP_s_, they have had untoward effects such as increased risk of arteriovascular disorders [6]. Accordingly, the current accomplished studies also indicate consuming OCP_s_ are associated with ischemic cardio-vascular injuries and also coagulopathy causing increased cardiovascular reactions and microalbominuria. Hence, the impacts of OCP_s_ are considered a significant problem both for the health care providers and also the users [7].

Cardiovascular diseases are considered as a public health issue with remarkable morbidities and mortalities and also economic burden. Therefore, determining the risk factors is a health priority. Several epidemiological studies have shown that old age [7], obesity, high blood pressure, diabetes [7, 8].

Smoking and high cholesterol level are among the risk factors for heart diseases. Hyperhomocysteinemia has been identified as one of the main risk factors for cardiovascular diseases since the past decade [7, 9].

Five micromole increase in total plasma level of homocysteine may increase the risk of developing coronary heart disease to 60–80%, cerebro-vascular disease to 50% and may cause 6- fold increased peripheral vascular diseases [10].

Homocysteine (HCY) is a sulfur- containing amino acid being synthesized by methionine metabolism as an intermediate solution [10].

Nitric oxide is synthesized in vessel endothelium by L- arginine [11] and it can cause vascular dilatation and prevents the accumulation of smooth muscle cells and platelets and their migration [12, 13].

This phenomenon can be as the result of abnormal reactions between vessel walls and platelets initiating and developing arteriosclerosis. Moreover, there are some evidences regarding endothelial dysfunction and the special role of NO; i.e. vasodilatation, in patients suffering from hypercholesterolemia, hypertension, Hyperhomocysteinemia, and in smokers [13–16].

Arteriosclerosis is the commonest form of coronary heart disease caused by gradual disposition of lipids and calcium in coronary arteries of heart muscles [17]. Epidemiological studies have demonstrated that exposure to OCP_s_ may change lipid metabolism [18, 19].

Additionally, it has been reported that OCP_s_ can change the concentrations of some plasma lipids including total cholesterol, high density lipoprotein (HDL-c), low density lipoprotein (LDL-c), and triglyceride [19]. Consuming OCP_s_ can have adverse effects on lipid profiles in healthy women, i.e. it can increase triglyceride levels and decrease HDL levels [20].

Although there have been conducted various researches about the effects of OCP_s_ on lipid profiles [19, 21–23], there have been controversies in the obtained results. As homocysteine is currently known as a cardiovascular disease risk factor affecting NO levels and given the limited carried out studies in this regard, the present research was accomplished to investigate the effects of exposure to low dose OCP on HCY levels, NO, lipid profiles being considered as cardiovascular disease risk factors in healthy women in the city of Yazd, Iran.

## Methods

This was a combined cohort study (retrospective+ prospective) conducted on 100 married women. The sample population consisted of married women at the age range of 20–30 years old settled in the city of Yazd, Iran. They referred to health care centers and family planning clinics. They had normal menstrual cycles.

The participants were divided into 2 groups: the exposed group to OCP_s_, and the non-exposed one. The first group encompassed the women referred to the center and they were administered LD OCP_s_ (manufactured by Aburayhan pharmaceutical company) at least for 3 days and at most for 36 days of a menstrual cycle. After having 21 pills, they stopped consuming the pills for 7 days.

After that, they took the next box of the pills. The second group included those women referred to the center for any reasons but they did not take any hormonal preparations of birth control.

The informed consent forms were obtained from the participants. Then, demographic data including age, occupation, educational level, family income level, the history of pregnancy, the type of delivery, the number of children, smoking and alcohol habits, the history of using drugs (e.g. OCP_s_), menstrual cycle, the duration time of OCP usage, and physical exercise were obtained through face to face interview.

In the study, the exposed and non-exposed participants were matched only regarding age in the form of frequency matching, so that non-exposed group were matched with exposed counterparts as 2 ± year(s).

The exclusion criteria included the history of repeated abortions (≥ 2 times) [24], thyroid dysfunction, the family history of heart diseases before the age of 40; the personal history of heart disease, diabetes, hepatic disease, renal dysfunction, dyslipidemia. Other exclusion criteria encompassed those working with pesticides, those having the habits of smoking by themselves or by the partners, drinking alcohol, the history of anemia; taking vitamin supplements such as folic acid, vitamins B6 and B12 during the last year, receiving a blood transfusion, and being pregnant during the previous year. The information was obtained from face to face interviews and was recorded on a checklist.

To determine the influencing factors on lipid profiles, homocysteine and nitric oxide levels, we studied some textbooks and essays in this literature. After consulting with the specialties, some influencing factors on blood biochemical parameters were determined and ultimately recorded and prepared as a checklist validated by the authorities. Based on the checklist, the candidates not having exclusion criteria were included the study.

Since the highest concentration of methionine is found in animal protein diets causing temporarily increased HCY levels which reaches to the maximum levels at 8 h after consuming the diets and lasts up to 24 h [25]; hence, the participants were advised not to have the diets containing animal proteins or any diets having the known impacts on NO and HCY levels like cheese, red meat, salad, spinach, canned foodstuff, and tea 24 h before blood sampling [13].

The participants were followed at least for 3 months. Then, the weight, blood pressure, BMI, and waist- hip ratio (WHR) were measured. Finally, the venous blood samples were taken from 2 groups and were sent to the central laboratory in Yazd to analyzing the biochemical parameters. The methods of the tests were measures base on standard methods [26–30].

The conditions and methods of blood biochemical parameter measurements: Being fasted for 12–14 h, the participants took part blood sampling. Ten milliliter of venous blood was taken from brachial veins of the participants of the groups.

To minimize the chemical changes, the samples were taken at 9 to 11 o’ clocks. Out of 10 ml, 4 ml was put into EDTA tubes and the rest (6 ml) in sodium citrate tubes being kept in ice through the sampling phase and till the samples were sent to the central laboratory of Yazd for analyzing the parameters.

The samples were centrifuged for 30–45 min. Then, total cholesterol, triglyceride, HDL-c and LDL-c levels were measured. Using auto Analyzer (Hitachi, Japan) and commercial kits manufactured by Bionic corporation being confirmed by Iran Health Reference Laboratory, triglyceride levels in serum by enzymatic method (lipase for converting triglyceride to glycerol), cholesterol levels by enzymatic cholesterol esterase (lipase for converting triglyceride to glycerol), HDL-c by enzymatic precipitation method were determined and LDL-c was calculated by Friedewald formula [31].

Homocysteine levels by the enzymatic and photometric method using homocysteine kit manufactured by Diazyme (Roche subsidiary company, USA), and using Auto Analyzer BT3000 (Biotechnics, Italy) were determined (coefficient of variation ≤5%). Nitric oxide levels were determined by photometric method using Greiss reaction where a micropipette reader (having CV < 5%), the kit manufactured by Iran Sib Bio, and ELISA microplate reader 3200 FaxStat (Awareness Technologies, USA) were used.

### Data analysis

Using SPSS software (version 16), statistical independent t-test, and chi square, the data were analyzed. Finally, the results obtained from the participants consuming the pills with the ones from those having non hormonal birth control methods were compared.

## Results

In the current study, 2 groups were similar regarding age, so that the age range for the exposed group was 30.12 ± 4.09 years, while it was 30.06 ± 4.06 for the non-exposed one.

The comparison between the mean and standard deviation regarding anthropometric indexes and blood pressures (BP_s_) for the groups is shown in Table 1. According to the Table 1, there were no significant differences concerning BMI, WHR, systolic and diastolic BP_s_ between two groups.

**Table 1:** Comparison of anthropometric indexes and BP_s_ between the two groups

Anthropometric indexes and BP	The users of LD pills	The non-users of LD pills	*P*- value
BMI (kg/ m2)	26.023 ± 3.935	25.356 ± 4.852	.045
WHR	0.847 ± 0.063	0.852 ± 0.073	0.71
Systolic BP (mmHg)	107.50 ± 9.978	105.22 ± 10.804	0.27
Diastolic BP (mmHg)	73.140 ± 7.331	71.04 ± 7.079	0.14

Table 2 demonstrates the mean and SD regarding homocysteine, nitric oxide levels and lipid profiles in the exposed women to LD pills vs. the non-exposed ones. As shown in Table 2, there were no significant statistical differences regarding the average of homocysteine and nitric oxide levels between the two groups. Additionally, there were no meaningful differences regarding HDL-c levels between the groups. But, there were comprehensible differences concerning the mean of cholesterol, LDL-c, and triglyceride levels between the groups.

**Table 2:** Comparison of homocysteine, nitric oxide levels and lipid profiles between the exposed women to LD pills vs. non-exposed ones

The risk factors of cardiovascular diseases	The users of LD pills (*n* = 50)	The non-users of LD pills (*n* = 50)	*P*- value
Homocysteine levels (μmol/l)	3.848 ± 2.357	3.284 ± 1.616	0.41
Nitric oxide (μM)	181.360 ± 90.44	162.654 ± 90.913	0.29
Cholesterol (mg/dl)	180.7 ± 38.28	159.74 ± 30.26	0.00
HDL-c (mg/dl)	56.46 ± 8.42	56.18 ± 8.91	0.87
LDL-c (mg/dl)	101.42 ± 30.66	84.84 ± 24.70	0.00
Triglyceride (mg/dl)	129.82 ± 47.92	93.60 ± 44.01	0.00

## Discussion

The current study investigated homocysteine, nitric oxide levels and lipid profiles in the exposed women to LD contraceptive pills versus the nonusers of hormonal birth control methods. There were no significant differences regarding anthropometric indexes between the groups. However, there were some controversies in this regard. A study carried out by Raf raf in Tabriz, Iran, demonstrated that there were no significant differences concerning the mean of BMI and the numbers of pregnancy between the OCP_s_ users versus the nonusers [32], although weight gaining has been reported as the untoward effects of OCP_S_, there have been limited experimental evidences confirming this notion [33].

A study fulfilled by Ainy in Tehran, Iran, manifested that there were no significant differences regarding the mean of systolic and diastolic BP_s_ between the groups [21].

The finding was probably due to the sample size of two studies. However, a study conducted by Wang C on Chinese women showed that using low dose OCP_s_ was associated with increased risk of hypertension [34] that might be contributed to general and abdominal obesity at the time of using the pills.

Based on the findings of the study, there were no statistically significant differences regarding LD contraceptive pills and the mean of homocysteine and nitric oxide levels in the women using the pills. It is known that homocysteine plasma level in pre-menopause stage and in pregnant women is lower than that in men and in post menopause stage [35].

Moreover, the level of this hormone is high during the luteal phase of menstruation period when steroid hormone levels are high, while it is low during the follicular phase when the levels of steroid hormones are low [36].

The studies have demonstrated that steroid hormones are regarded as non-genetic factors suggesting the theory that consuming LD pills is associated with homocysteine metabolism disorders. However, there have been reported contradicted results regarding untoward effects of the pills on homocysteine levels [16, 37].

A study carried out by Fallah et al., in Tehran, Iran, showed that the level of homocysteine was increased in the user of the pills and simultaneously the concentration of nitric oxide was significantly decreased [13].

The differences between the previous studies having the same sample size might be due to the inclusion and exclusion criteria. A study accomplished by Federico Lussana et al., in Italy being compatible with ours demonstrated that there were no meaningful differences concerning the plasma levels of homocysteine among the users of the pills being fasted or after consuming methionine orally in comparison with those levels among non-users [16].

A study fulfilled by Steegers-Theunissen demonstrated that consuming contraceptive pills increased the plasma levels of homocysteine in the users compared with the control group at the early stage of menstruation cycle (low hormone level) [38]. A study performed by Beaumont showed that consuming the pills increased the plasma levels of homocysteine in the women having the history of venous thromboembolism [39].

The findings were incompatible with ours; because in our study, blood sampling was not taken from the participants at the time of menstruation cycle. Additionally, our study was performed on healthy women and the reason of increased homocysteine level in the users of the pills having prior history of thromboembolism may be due to the known association between homocysteine and the risk of thromboembolism [40].

A study conducted by Gabriele et al. regarding the effect of gonadal hormones on nitric oxide production showed that 17-α estradiol increased endothelial NO production in cell cultures and also decreased nitric oxide synthesis (NOS) in mice aortic smooth muscle cells [40].

The discrepancies found in two studies can be elaborated by two reasons. Firstly, one study was conducted on animal model whose results were incompatible with the results obtained from human model. Secondly, the pills consumed by exposed women contained not only estradiol but also a combination of ethinyl estradiol and levonorgestrel. It is notable that some studies have shown that nitric oxide levels may be associated with the concentration of homocysteine. For example, a study accomplished by Kit Chow [14] demonstrated that increased level of homocysteine might decrease endothelial NO production due to oxidative mechanisms. On the other hand, it has been shown that treatment with homocysteine decreased glutathione peroxidase (GPX) activity yielding increased nitric oxide sensitivity to oxidative inactivation [40].

The obvious variations in homocysteine and nitric oxide levels in our study were incompatible with above mentioned studies. This may be as the result of limited sample size of the current study.

Based on other findings of the study, the pills increased the levels of total cholesterol, LDL-c, and triglyceride in the user women compared with those used non hormonal methods of birth control. There have been various proponent and opponent studies in this regard [18, 22, 23].

A study carried out by Naz in India showed comparable results with ours except for HDL level which the pills caused to be increased [19]. Unlike our study, a study fulfilled by Kisok Kim on Korean women showed that consuming OCPs increased HDL-c levels while it decreased LDL-c ones [18].

The difference may be due to the type of study, the sample size, the participants’ age range, racial diversities, and the sort of used pills. It is known that lipid metabolism disorders are considered significant risk factors for various diseases like cardiovascular ones [18]. According to the obtained results, consuming LD pills causing increased levels of cholesterol, triglyceride, and LDL-c may be a contributed risk factor for developing dyslipidemia.

There were some limitations in the present study. Firstly, having high cost and participants lost, the study was performed on a limited population whose some relations might be undetectable. Secondly, due to time limitation, the study was performed in a short period of time. Thirdly, irrespective of luteal and follicular phases of menstrual period, the participants underwent blood sampling only once. Fourthly, the governmental population planning was to encourage parents to have more children. Fifthly, the genetic parameter being affective on homocysteine level was excluded from the study due to time and cost limitations. And finally, being observational, the study had some limitations regarding the impacts of consuming foodstuff including cheese, red meat, salad, spinach, canned foods, and tea on homocysteine levels; therefore, the participants were asked not to consume the aforesaid foodstuff 24 h before blood sampling.

## Conclusion

Based on the obtained findings, consuming low dose contraceptive pills in the studied women caused no changes in the levels of homocysteine and nitric oxide being risk factors for developing cardiovascular diseases. Hence, consuming the pills in healthy women cannot develop cardiovascular diseases. Taking into account the importance of this matter, it is recommended that health providers administer the pills cautiously for the women: because as mentioned the study, the pills alter lipid metabolism. And also, it is recommended that health care providers always take into account the obtained results in their course of action. For example, when someone chooses to use the pills due to the cost-benefit and simplicity, it is advised to be followed up continuously to prevent lipid profile changes. If this condition occurs, the alternative methods should be substituted to prevent causing and developing cardiovascular diseases**.**

## Data Availability

Datasets are available through the corresponding author upon reasonable request.

## Abbreviations

LDL: Low Density Lipoprotein
HDL: High Density Lipoprotein
OCPs: Oral Contraceptive Pills
HCY: Homocysteine
NO: Nitric Oxide
WHR: Waist- Hip Ratio
BP_s_: Blood Pressures
BMI: Body Mass Index
